# Frequency analysis of Spinocerebellar ataxia types 1, 2, 3 & 6 in patients with ataxia from Gujarat

**DOI:** 10.1186/1755-8166-7-S1-P64

**Published:** 2014-01-21

**Authors:** Harsh Patel, Mehul Mistri, Chitra Ankleshwaria, Frenny Sheth, Jayesh Sheth

**Affiliations:** 1 Institute of Human Genetics, FRIGE House, Ahmedabad, Gujarat, India

## Background

The autosomal dominant spinocerebellar ataxias (ADCA) are a clinically and genetically heterogeneous group of neurodegenerative disorder characterized by progressive deterioration in balance and coordination as well as cerebellar ocular disturbance. There is a lack of information about the frequency of SCAs in Gujarat (western part of India), which can be used as a common screening test in our population. The study was conducted to analyze the frequencies of SCA1, SCA2, SCA3 and SCA6 in patients with ataxia from Gujarat.

## Materials and methods

Prospective analyses of 64 unrelated patients were included after an informed written consent. They were presented with progressive cerebellar ataxia with other features like gait and speech disturbance, saccadic eye movements and tremors. The study included 35 males and 29 females in the age range of 20 years to 85 years. All patients were investigated for CAG repeat expansion in SCA1, SCA2, SCA3 and SCA6 gene by PCR. Genomic DNA was used for the molecular study.

## Results

CAG repeat expansion was detected in 27 patients (42.1%) of 64 unrelated individuals. Among these, 7.41% (female-2) subjects were found to have SCA1 with CAG repeat expansion copy in the range of 49-56 in ATXN1 gene, 59.3% (female-9, male-7) were found to have SCA2 with CAG repeat expansion copy in the range of 37-47 in ATXN2 gene, 33.33% (female-3, male-9) were found to have SCA3 with CAG repeat expansion copy in the range of 63-82 in ATXN3 gene. While none of the subject was found to have SCA6 as has been shown by normal CAG repeats in the range of 10-16 in CACNA1A gene. Age range of onset of the affected subjects was 3^rd^ - 5^th^ decade in SCA1, 3^rd^ - 6^th^ decade in SCA2 and 3^rd^ - 8^th^ decade in SCA3.

## Conclusion

Our study demonstrates that SCA2 is the commonest dominant spinocerebellar ataxia in Gujarati population followed by SCA3 affecting in the 3^rd^ - 6^th^ decade of life.

